# Elevated Adiponectin Serum Levels in Women with Systemic Autoimmune Diseases

**DOI:** 10.1155/2010/938408

**Published:** 2010-12-26

**Authors:** Éric Toussirot, Béatrice Gaugler, Malika Bouhaddi, Nhu Uyen Nguyen, Philippe Saas, Gilles Dumoulin

**Affiliations:** ^1^Department of Rheumatology, University Hospital Jean Minjoz, Bd Fleming, 25030 Besançon Cedex, France; ^2^University Hospital St Jacques, CIC Biotherapy-CBT 506, 25030 Besançon Cedex, France; ^3^University of Franche Comté, Équipe d'Accueil 4266 “Agents Pathogènes et Inflammation”, IFR133, 25000 Besançon, France; ^4^INSERM UMR645, 1 Bd A Fleming, 25020 Besançon Cedex, France; ^5^University of Franche-Comté, UMR645, IFR133, 25020 Besançon Cedex, France; ^6^EFS Bourgogne Franche-Comté, Plateforme de Biomonitoring, 25020 Besançon Cedex, France; ^7^Department of Physiology, University Hospital Jean Minjoz, Bd Fleming, 25030 Besançon Cedex, France; ^8^University of Franche Comté, Équipe d'Accueil EA 3920 “Physiopathologie Cardiovasculaire et Prévention”, IFR133, 25000 Besançon, France

## Abstract

Adipose tissue produces a wide range of proteins that may influence the immune system. In this study, we assessed the serum levels of leptin, adiponectin, and ghrelin, in association with the measurements of body composition, in 15 female patients with various autoimmune diseases (systemic lupus erythematosus, primary Sjögren's syndrome, sarcoidosis, mixed connective tissue disease, vasculitis, CREST syndrome, and polymyositis) and in 15 healthy female controls. There were no statistically significant differences between the patients and controls with regard to serum leptin, serum ghrelin, global fat mass, adiposity, and fat mass in the android or gynoid regions, whereas serum adiponectin levels were higher in patients than controls (16.3 ± 1.6 *μ*g/mL versus 9.7 ± 0.6 *μ*g/mL; *P* = .01). As adiponectin is known to exhibit potent anti-inflammatory properties, a high adiponectinemia in patients with systemic autoimmune disease may mitigate the inflammatory response. However, the precise consequences of these elevated serum adiponectin levels on the metabolic syndrome development and atherosclerotic cardiovascular risk in this patient population still needs to be determined.

## 1. Introduction

It is now well established that the adipose tissue, and more precisely the white adipose tissue, is not only a storage organ but plays an active role in that it can produce and release various mediators that might play a role in the physiological processes [1, 2]. Indeed, adipocytes produce specific proteins or adipokines with a broad range of biological and physiological activities related to glucose homeostasis, lipid metabolism, appetite regulation, angiogenesis, hemostasis, and reproduction as well as immunity. Adipose tissue is composed of different cellular types, of which the adipocytes are the most abundant, but it also contains macrophages. These macrophages can release various cytokines including TNF*α* and IL-6 [1–3]. In obese patients, a low-grade systemic inflammation has been reported [1], as shown by increased CRP and IL-6 levels compared to those observed in lean subjects. This relationship between fat tissue and systemic inflammation reflects the adipose tissue's potential contribution with regard to the inflammatory response, which may be partly explained by the production of inflammatory cytokines. In fact, macrophages from adipose tissue have been shown to contribute to up to 30% of circulating IL-6, indicating that adipose tissue is a significant production site of circulating proinflammatory cytokines [1, 2].

The main adipokines studied were leptin and adiponectin. Leptin's role on appetite regulation is well documented, while adiponectin has been shown to be involved in insulin sensitivity [1, 2]. The influence of these adipokines on immunity and inflammation has been well documented. In most inflammatory diseases, it is generally accepted that leptin displays proinflammatory effects, while adiponectin is considered to primarily act as an anti-inflammatory molecule. As an example, it has been reported that leptin receptors are expressed on B and T-cells, and that leptin may exert direct effects on lymphocytes. Leptin may stimulate T cell proliferation, promote a Th1 response, influence T-cell activation, and activate macrophages and monocytes, thereby enhancing their phagocytic activities. In animal models, leptin deficiency has been associated with immune suppression [1–4]. Adiponectin exerts a variety of anti-inflammatory activities, interfering with macrophage function by inhibiting phagocytosis, IL-6 or TNF*α* production, reducing T-cell function, and promoting the release of IL-10 and IL-1 receptor antagonist [1–3, 5]. Taken together, adipose tissue may influence or modulate the inflammatory response.

Connective tissue diseases are systemic disorders which may have a potential impact on several organs, and result from the complex interaction between immunological, psychological, environmental, hormonal, and genetic predisposing factors. Systemic lupus erythematosus (SLE) and Sjögren's syndrome (SS) are two prototypical autoimmune diseases. This disease group also includes sarcoidosis, systemic sclerosis, dermato/polymyositis, and systemic vasculitis. A common feature of all of these disorders is the contributive and interactive role of autoantibodies, immune complexes, and soluble mediators of cellular communication (e.g., interleukins, chemokines, and growth factors) in tissue damage.

Serum leptin and adiponectin have been previously assessed in patients with SLE, giving conflicting results [6–11]. This study aimed to assess circulating leptin and adiponectin in patients with various systemic autoimmune diseases in parallel with the evaluation of body composition, and more especially fat mass. We also investigated serum levels of ghrelin, a gastric peptide involved in both appetite control and immunity regulation.

## 2. Patients and Methods

### 2.1. Patients

Fifteen white consecutive outpatients with systemic autoimmune disease who were seen in our department were evaluated. This patient series included only women, as it is well known that autoimmune diseases occur primarily in women. The study group comprised patients with SLE (*N* = 3), Sjögren's syndrome (*N* = 7), sarcoidosis (*N* = 1), polymyositis (*N* = 1), CREST syndrome (*N* = 1), mixed connective tissue disease (*N* = 1), or undifferentiated vasculitis (*N* = 1). All these patients met the specific diagnostic criteria of the respective systemic autoimmune diseases. Clinical assessment included demographic data such as age, body mass index (BMI), disease duration, and laboratory parameters of inflammation (erythrocyte sedimentation rate [ESR], and CRP and IL-6 levels). To be included in the study, no elevated acute phase reactants were required. Excluded from the study were patients with diabetes mellitus and other endocrine disorders (Cushing syndrome or thyroid disease).The treatments administered included nonsteroidal anti-inflammatory drugs and second-line treatments (hydroxychloroquine, methotrexate). Corticosteroid dosage was required to be below or equal to 10 mg prednisone per day; cumulative and mean corticosteroid dosages were also calculated. Among the patient group, 13 women were postmenopausal. As the patients suffered from various autoimmune diseases, no specific scoring system for clinical disease activity was used.

### 2.2. Controls

The control group included 15 healthy white women with no history of inflammatory condition, metabolic/endocrine disorders, or drug treatment (hospital staff). They were matched to the patients with regard to age and BMI. The exclusion criteria were the same as those for the patient group. Among the control group, 10 women were postmenopausal.

The study protocol was approved by our institutional ethics committee (*Comité d'Éthique Clinique du CHU de Besançon*).

### 2.3. Measurements of Adipose Tissue

A total body scan was performed using a Lunar iDXA densitometer (Lunar, Madison, WI, USA). Measurements for body composition were taken from the total body scan including fat mass and lean mass. Total and regional body fat mass and lean mass were also measured. Adiposity (% fat) was defined as the ratio between total fat tissue and total lean mass + total fat tissue. Fat distribution was evaluated as the relative proportion of fat tissue in the android (central) and the gynoid (hip and thigh) regions. In our hands, the coefficients of variation for values for adiposity, fat mass, and lean mass were 0.63%, 0.59%, and 0.45%, respectively, [12].

### 2.4. Adipokines, Ghrelin, and Insulin

After overnight fasting, venous blood samples were taken at 8.00 a.m. from each patient and control subject. The serum was stored at −20°C. Radioimmunoassay was used for measurements of leptin (Mediagnost GmbH, Reutlingen, Germany) as well as for total adiponectin (including high-molecular and low-molecular weight complexes) and total ghrelin (Linco Research, St Charles, MO, USA). Interassay coefficients of variation were 5.7% for leptin, 6.2% for adiponectin, and 7.9% for ghelin. The lowest level that could be detected by each assay (sensitivity) was 0.5 ng/mL for leptin, 1 *μ*g/mL for adiponectin, and 93 pg/mL for ghrelin. Serum leptin concentrations were adjusted for fat mass. In addition, fasting glycemia and insulin levels were determined (enzyme immunoassay, ST AIA-PACK IRI, Tosoh corporation, Tokyo, Japan). The homeostasis model assessment index for insulin resistance (HOMA-IR), used as a marker of insulin resistance, was calculated as previously described: fasting insulin (*μ*U/mL) × fasting glucose (mmol/L)/22.5 [13]. Detection of IL-6 in the patients' sera was performed by ELISA using IL-6 ELISA kit II BD OptEIA (BD Biosciences, Le Pont de Claix, France). Fasting serum total cholesterol as well as low-density lipoprotein (LDL) cholesterol and high-density lipoprotein (HDL) cholesterol were also evaluated.

### 2.5. Statistical Analysis

The data was analyzed using a computed package for statistical analysis (Alsyd, SAS, Meylan, France). All the measured variables were compared between study patients and controls using the nonparametric Mann-Whitney * U-*test . The relationship between the different variables (fat measurements, laboratory parameters of disease activity, and serum adipokines and ghrelin levels) were analyzed using the Spearman's *r* test. A *P* value inferior to .05 was considered statistically significant.

## 3. Results

The results were expressed as mean ± SEM, as shown in Table 1. In the whole series, five patients and three healthy controls were considered obese (BMI ≥ 30 kg/m^2^). Overall, 11 patients were receiving prednisone at a mean dosage of 6.6 ± 1.3 mg /day, while six were taking hydroxychloroquine and seven methotrexate at study initiation. The cumulative corticosteroid dosage for these patients was 11018 ± 4481 mg prednisone equivalent. 

**Figure 1:**
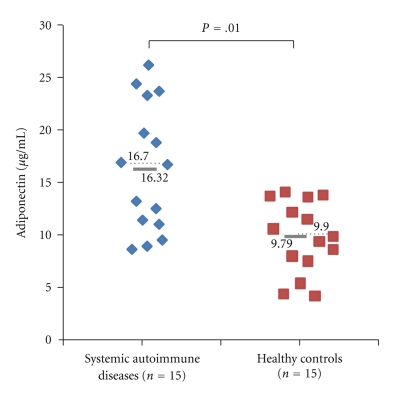
Serum adiponectin levels in 15 women suffering from systemic autoimmune disease and in 15 healthy women. Each value is represented by a blue diamond (patients with systemic autoimmune disease) or a red square (control women). Means and medians are represented by a grey bold line and black dotted line, respectively. Mean and median values for each group are also given. For statistical comparison, the Mann-Whitney *U*-test was used.

As expected, patients and controls did not differ regarding age and BMI. There were no significant between-group differences in erythrocyte sedimentation rate and CRP levels, while IL-6 serum levels were markedly elevated in the patient group (9.02 ± 3.7 pg/mL versus 0.02 ± 0.02 pg/mL; *P* = .025). Between both groups, we found no significant differences regarding fat mass and lean mass as well as adiposity and fat distribution in the android and gynoid regions (all *P* > .05). Adiponectin levels were higher in patients with systemic autoimmune disease than in control subjects (16.3 ± 1.6 *μ*g/mL versus 9.7 ± 0.6 *μ*g/mL; *P* = .01) (Figure 1). Conversely, no statistically significant differences between patients and controls were observed for fasting glycemia, insulin levels, HOMA-IR, total serum cholesterol, LDL and HDL cholesterol, ghrelin, leptin, and leptin corrected by fat mass (all *P* > .05). 

The relationships between fat mass, BMI, ghrelin, adipokines, and disease activity parameters were then examined. As expected, we found a strong correlation between circulating leptin levels and BMI, fat mass, or adiposity in both patients (*r* = 0.92, *r* = 0.91, and *r* = 0.88, resp.; *P* < .001 for all tests) and control subjects (*r* = 0.93, *r* = 0.91 and *r* = .95, resp.; *P* < .001 for all tests). By contrast, and as previously described [1, 2], there was no direct correlation between adiponectin or ghrelin and fat mass measurements. Body mass index, fat mass, and adiposity were slightly correlated with CRP levels (*r* = 0.41, *r* = 0.49, and *r* = 0.45, resp.; *P* ≤ .05 for all tests) but not with ESR or IL-6. Additionally, the relationships between disease activity markers and serum adiponectin levels were examined, and ESR and adiponectin were found to be markedly and negatively correlated (*r* = −  0.8, *P* = .003), whereas no relationship was observed between adiponectin and CRP or IL-6 levels. 

The serum levels of certain adipokines may be influenced by some treatments including corticosteroids and methotrexate. For this reason, adiponectin serum levels in the patient group were examined in relation with corticosteroid or methotrexate use. Although adiponectin serum levels were found to be higher in patients with or without corticosteroids as compared to healthy controls, the difference reached statistical significance only for patients who were not taking corticosteroids (healthy subjects (*N* = 15) versus autoimmune disease patients without corticosteroids (*N* = 4): 10.64 ± 0.8 *μ*g/mL versus 19.9 ± 3.3 *μ*g/mL; *P* = .02), whereas only a trend towards a higher adiponectinemia was observed for patients who were actually taking corticosteroids (healthy subjects (*N* = 15) versus autoimmune disease patients with corticosteroids (*N* = 11): 10.64 ± 0.8 *μ*g/mL versus 15.1 ± 1.7 *μ*g/mL; *P* = .07). Similar results were found for adiponectin when examining the use of methotrexate (healthy subjects (*N* = 15) versus patients without methotrexate (*N* = 8): 10.64 ± 0.8 *μ*g/mL versus 17.2 ± 2.4 *μ*g/mL; *P* = .04; healthy subjects (*N* = 15) versus patients with methotexate (*N* = 7): 10.64 ± 0.8 *μ*g/mL versus 15.2 ± 2.1 *μ*g/mL; *P* =  .07). 

As our series included mainly subjects with Sjögren's syndrome (*N* = 7), results from this subgroup were analyzed separately and compared to those obtained in healthy subjects (Table 1). Similarly to the entire patient group, higher IL-6 and adiponectin serum levels were noted in patients with Sjögren's syndrome as compared to healthy controls (*P* = .05 and *P* = .04, resp.).

## 4. Discussion

In this study, we assessed the serum levels of adipokines such leptin, adiponectin, and ghrelin, in association with body composition measurements, in women with various systemic autoimmune diseases. The patient data was compared to the results obtained in healthy subjects. The main outcome of our study was that patients with systemic autoimmune disease presented higher adiponectin levels than control subjects, in contrast with the absence of differences observed in serum leptin and ghrelin levels. Similarly, measurements of body composition and distribution did not differ between both groups. Although patients with various autoimmune diseases were included in our study, they were carefully matched for age, sex, and BMI with the control subjects in order to obtain a homogeneous comparison group. In addition, all systemic autoimmune diseases included in the study were characterized by common pathophysiological processes induced by inflammatory mediators, that is, cytokines such as TNF*α* or IL-6, and adipokines as well. As our patient population was mainly comprised of patients with primary Sjögren's syndrome (*N* = 7), this prompted us to analyze, separately, the results for this patient subgroup. In accordance with the data gathered in the entire patient group, high adiponectinemiae (and IL-6 as well) was observed in this subgroup. To our knowledge, no study so far has evaluated adipokine levels in this specific patient population. 

Our patients were receiving corticosteroid treatments at a mean daily dose of 6.6 ± 1.3 mg prednisone equivalent. Corticosteroid treatment has been associated with the development of adiposity involving predominantly the body's central or visceral regions, and contributing to increased cardiovascular risk [14]. However, there was no difference in android or visceral fat mass between the patients and controls participating in our study. This may be explained by the fact that our patients had received low-dose corticosteroid therapy. An interesting observation of our study was that there was a significant relationship between fat mass measurements (total fat mass, adiposity) and laboratory inflammation parameters such as CRP, reflecting the potential contribution of the adipose tissue to the inflammatory response. 

Our patients exhibited a significant elevation in serum adiponectin levels in line with previous works on SLE patients [7, 8, 11], but no data was available so far on adiponectin levels in patients suffering from other systemic autoimmune diseases, notably primary Sjögren's syndrome. The high adiponectinemia found in our patient series cannot be explained by changes in fat mass or fat distribution. Our patients were taking corticosteroids and/or methotrexate, and these treatments have been associated with changes in circulating adiponectin levels. However, high adiponectinemia was observed in both patients receiving or not receiving corticosteroids or methotrexate, with statistically significant results observed only in the subgroup that was receiving these drugs. While the effects of corticosteroids on serum adiponectin levels are controversial, [15, 16] adiponectin has been shown to increase modestly (13%) during methotrexate treatment in rheumatoid arthritis (RA) [17]. 

Previous studies have already reported high adiponectin levels in systemic autoimmune diseases, even in patients with a marked inflammatory response [8]. As proinflammatory factors such as cytokines are known to suppress adiponectin production by adipocytes, this high adiponectinemia in patients with inflammatory conditions such systemic autoimmune diseases is paradoxical. Taking into account adiponectin's anti-inflammatory properties, the results of our study can be interpreted as a dampening effect of adiponectin on inflammation [5]. The negative correlation that we noted between adiponectin and ESR supports this hypothesis, suggesting that a rise in adiponectin levels could be a mechanism involved in the control of inflammatory or immunological processes occurring in these disease states. Alternatively, adiponectin has been described as a potent proinflammatory mediator, as it has been shown to stimulate the production of IL-6 and prostaglandin E2 by synovial fibroblasts in rheumatoid arthritis patients, with serum adiponectin levels correlating to the severity of rheumatoid arthritis evaluated by the extent of joint destruction [18–20]. Thus, depending on the model, adiponectin has been reported to display anti- or proinflammatory activities. However, based on literature data, adiponectin appears to display a predominantly anti-inflammatory activity. 

 In addition to its pro/anti-inflammatory properties, adiponectin has emerged as a key determinant of the metabolic syndrome and may be considered as a protective agent against cardiovascular risk [21]. Indeed, adiponectin levels have been reported to be reduced in patients presenting type 2 diabetes or metabolic syndrome, and low plasma adiponectin concentrations closely correlate with obesity-related diseases such as atherosclerotic cardiovascular diseases [3, 21]. SLE has been shown to be associated with insulin resistance, metabolic syndrome, and high cardiovascular risk [7–9]. Unexpectedly, however, adiponectinemia was found to be elevated in patients with SLE and insulin resistance [7]. In our patients, we did not specifically evaluate all of the components of the metabolic syndrome, but high adiponectinemia did not influence insulin levels, insulin resistance, or changes in lipid profile. Whether high adiponectinemia could have clinical consequences in these patients, especially with regard to cardiovascular disease risk, would require further longitudinal analysis. 

Leptin serum levels have been evaluated in various inflammatory conditions [2]. In RA, circulating and synovial leptin levels were reported to be elevated compared to those observed in healthy controls or osteoarthritis patients [2]. In SLE, elevated serum leptin levels were observed in most studies [6–9], while some authors reported normal or decreased serum levels [10, 11]. On the basis of the results observed in SLE patients, the authors speculated on the possible influence of leptin on inflammation, certain clinical manifestations, or weight changes during the course of the disease [7]. However, no correlation was found between leptin levels and clinical or biological disease activity indices [8]. Our series included only three patients with SLE, and these patients did not exhibit changes in circulating leptin. 

Ghrelin was also evaluated in this study. This gastric peptide has several physiologic functions involving GH secretion, gastric function, control of blood pressure, and adiposity as well as appetite stimulation and decreased fat utilization. In addition to its role in the energetic balance, ghrelin exhibits anti-inflammatory properties and therefore, its determination is relevant in studies investigating adipokine levels in inflammatory disease states. Ghrelin has been shown to modulate phagocytosis, while downregulating innate immunity [22, 23]. Ghrelin receptors are expressed on human monocytes, and the binding of ghrelin to these receptors inhibits the production of proinflammatory cytokines such as IL-1, IL-6, or TNF*α* [24]. Ghrelin has previously been evaluated in patients with RA [24]. While decreased ghrelin serum levels were reported in RA patients, circulating ghrelin was increased in patients suffering from ankylosing spondylitis, another inflammatory condition [25]. These variations in ghrelin levels may influence the different inflammatory responses. However, our case series did not exhibit significant changes in serum ghrelin levels. 

In conclusion, our study did not reveal any significant differences in serum leptin and ghrelin levels as well as in body composition and distribution between systemic autoimmune disease patients and healthy controls. Contrarily, patients with systemic autoimmune disease, particularly Sjögren's syndrome, exhibited a marked increase in circulating adiponectin. As adiponectin is thought to mainly exert anti-inflammatory activities, high adiponectin levels could influence or mitigate the inflammatory response or immunopathological mechanisms involved in these disease states. However, the precise consequences of this high adiponectinemia on the metabolic syndrome development and atherosclerotic cardiovascular risk in this patient population still need to be determined.

## Figures and Tables

**Table 1:** Demographics, body composition and distribution, serum adipokines and ghrelin levels in women with systemic autoimmune disease, in patients with Sjögren syndrome and healthy women (results are given as mean ± SEM; *P* ^1^: Man Whitney test between patients with systemic autoimmune disease and healthy controls; *P* ^2^: Man Whitney test between patients with Sjögren's syndrome and healthy controls).

	Systemic autoimmune diseases whole series (*N* = 15)	Sjögren syndrome (*N = 7*)	Healthy controls (*N = 15*)	*P* ^1^	*P* ^2^
Age (years)	57.7 ± 3.5	59.5 ± 3.7	54.5 ± 2.1	NS	NS
Disease duration (years)	6.5 ± 1.4	4.7 ± 1.1	—	—	—
Clinical manifestations	Polyarthritis/arthralgia: *N* = 14 Myalgia *N* = 2 Dermatological manifestations *N* = 2 Sicca syndrome *N* = 7 Peripheral neuropathy *N* = 1 Pulmonary involvement *N* = 2 Raynaud syndrome *N* = 3 Renal disease *N* = 0	—	—	—	—
BMI (kg/m^2^)	29.0 ± 1.9	32.03 ± 3.5	25.4 ± 1.1	NS	NS
Corticosteroid dosage (mg/day)	6.6 ± 1.3	5.7 ± 2.2	—	—	—
Cumulative corticosteroid dose (mg)	11018 ± 4481	9192.8 ± 5349	—	—	—
ESR (mm/H)	16.3 ± 2.6	17.8 ± 4.6	14.5 ± 5.3	NS	NS
CRP (mg/L)	6.5 ± 1.9	5 ± 2	5.7 ± 1.8	NS	NS
IL-6 (pg/mL)	9.02 ±3.69	6.7 ± 3.2	0.02 ± 0.02	.025	.05
Glycemia (mmol/L)	4.8 ± 0.1	4.9 ± 0.2	4.6 ± 0.1	NS	NS
Insulin (*μ*IU/L)	10.3 ± 2.05	12.8 ± 3.9	8.6 ± 1.1	NS	NS
HOMA-IR	2.14 ± 0.37	2.6 ± 0.6	1.49 ± 0.15	NS	NS
Total cholesterol (g/L)	1.88 ± 0.1	1.9 ± 0.2	1.83 ± 0.1	NS	NS
LDL cholesterol (g/L)	1.02 ± 0.1	1.05 ± 0.2	1.03 ± 0.1	NS	NS
HDL cholesterol (g/L)	0.61 ± 0.04	0.7 ± 0.05	0.57 ± 0.04	NS	NS
Leptin (ng/mL)	25.2 ± 5.6	31.1 ± 10.6	14.4 ± 3.4	NS	NS
Leptin /fat mass (ng/mL/Kg)	0.72 ± 0.1	0.7 ± 0.2	0.82 ± 0.1	NS	NS
Adiponectin (*μ*g/mL)	16.3 ± 1.6	17.8 ± 2.6	9.7 ± 0.6	.01	.04
Ghrelin (pg/mL)	1118 ± 127	1249. 1 ± 220.5	1012 ± 56	NS	NS
Fat mass (g)	30140 ± 3411	33554.1 ± 5573.1	21518 ± 1862	NS	NS
Lean mass (g)	40822 ± 1348	42475.7 ± 2128.5	47011 ± 1908	NS	NS
Adiposity (%)	40.9 ± 2.3	42.8 ± 2.8	30.8 ± 1.8	NS	NS
Fat in the android region (g)	2681 ± 421	3099 ± 741.1	1951 ± 222	NS	NS
Fat in the gynoid region (g)	5710 ± 541	6282.9 ± 794.2	3905 ± 340	NS	NS

(ESR: erythrocyte sedimentation rate; BMI: body mass index; HOMA-IR: homeostasis model assessment index; NS: nonsignificant).
